# {(+)-(1*R*,2*R*)-1,2-Diphenyl-2,2′-[ethane-1,2-diylbis(nitrilo­methyl­idyne)]­di­phenol­ato}dipyridine­cobalt(III) perchlorate sesquihydrate

**DOI:** 10.1107/S1600536809002293

**Published:** 2009-01-23

**Authors:** Lian-Wen Zhou

**Affiliations:** a Department of Chemistry, Dezhou University, Dezhou 253023, People’s Republic of China

## Abstract

In title complex, [Co(C_28_H_22_N_2_O_2_)(C_5_H_5_N)_2_]ClO_4_·1.5H_2_O, the Co^III^ ion is in a slightly distorted octa­hedral coordination environment with the pyridine ligands in a *trans* arrangement. In addition to the cation and anion, the asymmetric unit also contains three half-occupancy solvent water mol­ecules and all components are connected *via* inter­molecular O—H⋯O hydrogen bonds.

## Related literature

For background information, see: Amirnasr *et al.* (2001 ▶); Cmi *et al.* (1998 ▶); Polson *et al.* (1997 ▶); Yamada (1999 ▶); Henson *et al.* (1999 ▶). For the synthethis of the parent Schiff base ligand, see: Zhang *et al.* (1990 ▶). For a related structure, see: Shi *et al.* (1995 ▶).
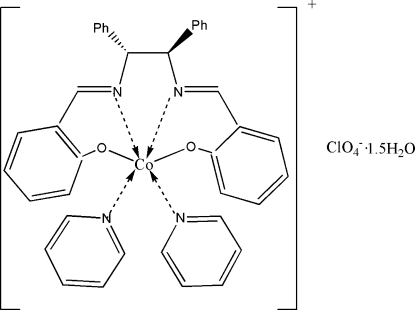


## Experimental

### 

#### Crystal data

[Co(C_28_H_22_N_2_O_2_)(C_5_H_5_N)_2_]ClO_4_·1.5H_2_O
*M* *_r_* = 762.08
Orthorhombic, 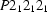

*a* = 10.9214 (6) Å
*b* = 18.3856 (10) Å
*c* = 18.6714 (11) Å
*V* = 3749.2 (4) Å^3^
*Z* = 4
Mo *K*α radiation
μ = 0.58 mm^−1^
*T* = 293 (2) K
0.21 × 0.16 × 0.13 mm

#### Data collection

Bruker APEXII CCD area-detector diffractometer
Absorption correction: multi-scan (*SADABS*; Sheldrick, 1996 ▶) *T*_min_ = 0.887, *T*_max_ = 0.928
20101 measured reflections
7290 independent reflections
4733 reflections with *I* > 2σ(*I*)
*R*_int_ = 0.059

#### Refinement

*R*[*F* ^2^ > 2σ(*F* ^2^)] = 0.050
*wR*(*F* ^2^) = 0.127
*S* = 0.99
7290 reflections
480 parameters
H-atom parameters constrained
Δρ_max_ = 0.51 e Å^−3^
Δρ_min_ = −0.29 e Å^−3^
Absolute structure: Flack (1983 ▶), with 3227 Friedel pairs
Flack parameter: 0.02 (2)

### 

Data collection: *APEX2* (Bruker, 2007 ▶); cell refinement: *SAINT-Plus* (Bruker, 2007 ▶); data reduction: *SAINT-Plus*; program(s) used to solve structure: *SHELXS97* (Sheldrick, 2008 ▶); program(s) used to refine structure: *SHELXL97* (Sheldrick, 2008 ▶); molecular graphics: *SHELXTL* (Sheldrick, 2008 ▶); software used to prepare material for publication: *SHELXTL*.

## Supplementary Material

Crystal structure: contains datablocks global, I. DOI: 10.1107/S1600536809002293/lh2754sup1.cif

Structure factors: contains datablocks I. DOI: 10.1107/S1600536809002293/lh2754Isup2.hkl

Additional supplementary materials:  crystallographic information; 3D view; checkCIF report

## Figures and Tables

**Table 1:** Selected bond lengths (Å)

Co1—O2	1.881 (3)
Co1—O1	1.889 (3)
Co1—N2	1.897 (3)
Co1—N1	1.904 (3)
Co1—N4	1.973 (4)
Co1—N3	1.978 (4)

**Table 2:** Hydrogen-bond geometry (Å, °)

*D*—H⋯*A*	*D*—H	H⋯*A*	*D*⋯*A*	*D*—H⋯*A*
O7—H7*C*⋯O8	0.85	1.85	2.701 (12)	176
O7—H7*D*⋯O1^i^	0.85	2.04	2.888 (8)	176
O8—H8*C*⋯O9	0.85	1.73	2.575 (14)	177
O8—H8*D*⋯O4^ii^	0.85	1.99	2.835 (10)	176
O9—H9*C*⋯O3^ii^	0.85	2.36	3.175 (11)	161

Symmetry codes: (i) 
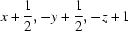
; (ii) 
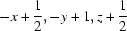
.

**Fig. 1.**
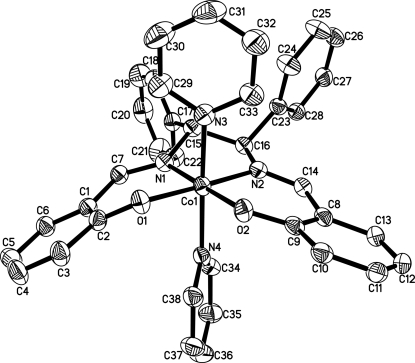
The cation of the title complex with the atom-labelling scheme. Displacement ellipsoids are drawn at the 30% probability level. H atoms are not shown.

## supplementary crystallographic information

### Comment

Cobalt complexes with tetradentate Schiff base ligands have been extensively used to mimic cobalamin (B_12_) coenzymes (Amirnasr *et al.*, 2001; Cmi *et al.*, 1998; Polson *et al.*, 1997) and dioxygen carriers and oxygen activators (Yamada, 1999; Henson *et al.*, 1999). Here, we report the crystal structure of a Co^III^ complex containing the chiral tetradentate Schiff base ligand (+)-(1*R*,2*R*)-*N*,*N*'-Bis(salicylidene)-1,2-diphenyl-1,2-ethanediamine.

The molecular structure of the title cation is shown in Fig. 1. The Co^III^ ion is six coordinated. The four equational sites are occupied by two N atoms and two O atoms of the tetradentate Schiff base ligand and the two axial sites are occupied by the N atoms of two pyridine ligands, forming a slightly distorted octahedral coordination environment. The Co—O and Co—N_Schiff base_ bond lengths are consistent with the corresponding bond lengths in the Co^III^ Schiff base complex *trans*-[Co(salen)(py)_2_][BPh_4_] (Shi *et al.*, 1995) as are the Co—N_py_ distances.

### Experimental

The free Schiff base ligand (*L*), it was prepared according to the method reported previously (Zhang *et al.*, 1990). The synthesis of the title complex was carried out by mixing CoClO_4_.6H_2_O, pyridine and *L* with a molar ratio 1:2:1 in methanol. After the mixture was stirred for about 30 min at room temperature in air, it was filtered to remove any undissolved material. The filtrate was allowed to partially evaporate in air for several days to produce crystals suitable for X-ray diffraction with a yield about 40%.

### Refinement

H atoms bonded to O atoms were located in a difference Fourier map. They were refined in a riding-model approximation with O—H = 0.85 Å and *U*_iso_(H) = 1.2*U*_eq_(O). H atoms bonded to C atoms were placed in calculated positions with C—H distances = 0.93 and 0.98 Å, and were refined in a riding-model approximation with *U*_iso_(H) = 1.2*U*_eq_(C).

### Crystal data

**Table:**

[Co(C_28_H_22_N_2_O_2_)(C_5_H_5_N)_2_]ClO_4_·1.5H_2_O	*F*(000) = 1580
*M**_r_* = 762.08	*D*_x_ = 1.350 Mg m^−^^3^*D*_m_ = 1.35 Mg m^−^^3^*D*_m_ measured by not measured
Orthorhombic, *P*2_1_2_1_2_1_	Mo *K*α radiation, λ = 0.71073 Å
Hall symbol: P 2ac 2ab	Cell parameters from 2979 reflections
*a* = 10.9214 (6) Å	θ = 2.4–20.0°
*b* = 18.3856 (10) Å	µ = 0.58 mm^−^^1^
*c* = 18.6714 (11) Å	*T* = 293 K
*V* = 3749.2 (4) Å^3^	Block, red-brown
*Z* = 4	0.21 × 0.16 × 0.13 mm

### Data collection

**Table:**

Bruker APEXII CCD area-detector diffractometer	7290 independent reflections
Radiation source: fine-focus sealed tube	4733 reflections with *I* > 2σ(*I*)
graphite	*R*_int_ = 0.059
φ and ω scans	θ_max_ = 26.0°, θ_min_ = 2.2°
Absorption correction: multi-scan (*SADABS*; Sheldrick, 1996)	*h* = −12→13
*T*_min_ = 0.887, *T*_max_ = 0.928	*k* = −20→22
20101 measured reflections	*l* = −22→23

### Refinement

**Table:**

Refinement on *F*^2^	Secondary atom site location: difference Fourier map
Least-squares matrix: full	Hydrogen site location: inferred from neighbouring sites
*R*[*F*^2^ > 2σ(*F*^2^)] = 0.050	H-atom parameters constrained
*wR*(*F*^2^) = 0.127	*w* = 1/[σ^2^(*F*_o_^2^) + (0.0572*P*)^2^] where *P* = (*F*_o_^2^ + 2*F*_c_^2^)/3
*S* = 0.99	(Δ/σ)_max_ = 0.001
7290 reflections	Δρ_max_ = 0.51 e Å^−^^3^
480 parameters	Δρ_min_ = −0.29 e Å^−^^3^
0 restraints	Absolute structure: Flack (1983), with 3227 Friedel pairs
Primary atom site location: structure-invariant direct methods	Flack parameter: 0.02 (2)

### Special details

**Table:**

Geometry. All e.s.d.'s (except the e.s.d. in the dihedral angle between two l.s. planes) are estimated using the full covariance matrix. The cell e.s.d.'s are taken into account individually in the estimation of e.s.d.'s in distances, angles and torsion angles; correlations between e.s.d.'s in cell parameters are only used when they are defined by crystal symmetry. An approximate (isotropic) treatment of cell e.s.d.'s is used for estimating e.s.d.'s involving l.s. planes.
Refinement. Refinement of *F*^2^ against ALL reflections. The weighted *R*-factor *wR* and goodness of fit *S* are based on *F*^2^, conventional *R*-factors *R* are based on *F*, with *F* set to zero for negative *F*^2^. The threshold expression of *F*^2^ > σ(*F*^2^) is used only for calculating *R*-factors(gt) *etc*. and is not relevant to the choice of reflections for refinement. *R*-factors based on *F*^2^ are statistically about twice as large as those based on *F*, and *R*- factors based on ALL data will be even larger.

### Fractional atomic coordinates and isotropic or equivalent isotropic displacement parameters (Å^2^)

**Table:**

	*x*	*y*	*z*	*U*_iso_*/*U*_eq_	Occ. (<1)
Co1	0.24611 (5)	0.00305 (3)	0.17215 (3)	0.03757 (15)	
Cl1	0.35527 (13)	0.30536 (8)	0.25408 (9)	0.0736 (4)	
O1	0.1518 (3)	−0.08004 (15)	0.19449 (15)	0.0469 (8)	
O2	0.1443 (3)	0.01550 (15)	0.09181 (14)	0.0437 (7)	
O3	0.3774 (4)	0.24263 (19)	0.2999 (3)	0.0960 (14)	
O4	0.2856 (5)	0.3577 (2)	0.2928 (3)	0.1116 (16)	
O5	0.4678 (4)	0.3364 (2)	0.2330 (3)	0.0932 (14)	
O6	0.2887 (4)	0.2832 (3)	0.1939 (3)	0.1289 (19)	
O7	0.5661 (8)	0.6832 (4)	0.9108 (4)	0.088 (3)	0.50
H7C	0.4884	0.6827	0.9136	0.106*	0.50
H7D	0.5880	0.6522	0.8796	0.106*	0.50
O8	0.3200 (8)	0.6862 (5)	0.9246 (4)	0.103 (3)	0.50
H8C	0.2789	0.7231	0.9380	0.124*	0.50
H8D	0.2910	0.6716	0.8849	0.124*	0.50
O9	0.1910 (8)	0.7977 (6)	0.9603 (5)	0.123 (4)	0.50
H9C	0.1551	0.7868	0.9214	0.148*	0.50
H9D	0.1378	0.8014	0.9933	0.148*	0.50
N1	0.3507 (3)	−0.00967 (18)	0.25272 (17)	0.0388 (8)	
N2	0.3455 (3)	0.08553 (18)	0.15341 (17)	0.0367 (8)	
N3	0.3501 (4)	−0.0582 (2)	0.1098 (2)	0.0449 (9)	
N4	0.1290 (3)	0.06249 (19)	0.22734 (19)	0.0407 (9)	
C1	0.2065 (4)	−0.0821 (2)	0.3196 (2)	0.0408 (10)	
C2	0.1272 (4)	−0.0988 (2)	0.2617 (2)	0.0428 (11)	
C3	0.0206 (5)	−0.1379 (3)	0.2778 (3)	0.0560 (13)	
H3	−0.0320	−0.1509	0.2408	0.067*	
C4	−0.0077 (5)	−0.1571 (3)	0.3452 (3)	0.0629 (15)	
H4	−0.0801	−0.1823	0.3537	0.076*	
C5	0.0679 (5)	−0.1406 (3)	0.4024 (3)	0.0601 (14)	
H5	0.0466	−0.1538	0.4489	0.072*	
C6	0.1756 (4)	−0.1041 (2)	0.3888 (3)	0.0490 (12)	
H6	0.2287	−0.0938	0.4264	0.059*	
C7	0.3216 (4)	−0.0458 (2)	0.3094 (2)	0.0412 (11)	
H7	0.3789	−0.0486	0.3461	0.049*	
C8	0.2062 (4)	0.1393 (2)	0.0670 (2)	0.0421 (11)	
C9	0.1321 (4)	0.0775 (3)	0.0570 (2)	0.0403 (10)	
C10	0.0392 (4)	0.0813 (3)	0.0046 (2)	0.0480 (12)	
H10	−0.0084	0.0406	−0.0050	0.058*	
C11	0.0183 (4)	0.1454 (3)	−0.0326 (2)	0.0538 (13)	
H11	−0.0443	0.1472	−0.0663	0.065*	
C12	0.0887 (5)	0.2064 (3)	−0.0203 (3)	0.0511 (13)	
H12	0.0726	0.2492	−0.0451	0.061*	
C13	0.1821 (4)	0.2038 (3)	0.0282 (2)	0.0472 (12)	
H13	0.2303	0.2448	0.0358	0.057*	
C14	0.3136 (4)	0.1386 (3)	0.1134 (2)	0.0408 (11)	
H14	0.3626	0.1799	0.1139	0.049*	
C15	0.4731 (4)	0.0235 (2)	0.2419 (2)	0.0394 (10)	
H15	0.5198	−0.0089	0.2104	0.047*	
C16	0.4541 (4)	0.0942 (2)	0.2025 (2)	0.0377 (10)	
H16	0.4319	0.1309	0.2384	0.045*	
C17	0.5483 (4)	0.0344 (2)	0.3105 (2)	0.0432 (11)	
C18	0.6548 (4)	−0.0041 (3)	0.3210 (3)	0.0573 (11)	
H18	0.6797	−0.0381	0.2871	0.069*	
C19	0.7252 (5)	0.0073 (4)	0.3816 (3)	0.0746 (15)	
H19	0.7963	−0.0196	0.3886	0.089*	
C20	0.6908 (6)	0.0581 (4)	0.4313 (4)	0.0787 (19)	
H20	0.7392	0.0662	0.4715	0.094*	
C21	0.5833 (6)	0.0977 (3)	0.4217 (3)	0.0755 (17)	
H21	0.5593	0.1321	0.4553	0.091*	
C22	0.5124 (5)	0.0850 (3)	0.3614 (3)	0.0587 (14)	
H22	0.4400	0.1107	0.3550	0.070*	
C23	0.5701 (4)	0.1209 (2)	0.1656 (2)	0.0417 (10)	
C24	0.6226 (5)	0.0834 (3)	0.1097 (3)	0.0610 (14)	
H24	0.5848	0.0419	0.0918	0.073*	
C25	0.7327 (5)	0.1077 (3)	0.0797 (3)	0.0687 (15)	
H25	0.7678	0.0826	0.0417	0.082*	
C26	0.7883 (5)	0.1682 (3)	0.1062 (3)	0.0606 (15)	
H26	0.8616	0.1840	0.0862	0.073*	
C27	0.7379 (5)	0.2061 (2)	0.1619 (3)	0.0544 (12)	
H27	0.7770	0.2472	0.1798	0.065*	
C28	0.6272 (4)	0.1827 (2)	0.1917 (2)	0.0439 (11)	
H28	0.5919	0.2087	0.2290	0.053*	
C29	0.3837 (5)	−0.1252 (3)	0.1278 (3)	0.0616 (15)	
H29	0.3636	−0.1419	0.1733	0.074*	
C30	0.4459 (7)	−0.1706 (3)	0.0833 (4)	0.088 (2)	
H30	0.4667	−0.2174	0.0978	0.105*	
C31	0.4777 (6)	−0.1458 (4)	0.0160 (4)	0.087 (2)	
H31	0.5205	−0.1756	−0.0155	0.104*	
C32	0.4457 (6)	−0.0779 (4)	−0.0031 (3)	0.0739 (17)	
H32	0.4680	−0.0599	−0.0478	0.089*	
C33	0.3795 (5)	−0.0349 (3)	0.0439 (3)	0.0547 (13)	
H33	0.3548	0.0112	0.0294	0.066*	
C34	0.1608 (4)	0.1158 (2)	0.2719 (2)	0.0487 (12)	
H34	0.2436	0.1256	0.2785	0.058*	
C35	0.0761 (5)	0.1572 (3)	0.3087 (3)	0.0648 (15)	
H35	0.1015	0.1944	0.3391	0.078*	
C36	−0.0459 (5)	0.1425 (3)	0.2998 (3)	0.0732 (16)	
H36	−0.1049	0.1686	0.3249	0.088*	
C37	−0.0793 (5)	0.0884 (3)	0.2533 (3)	0.0661 (15)	
H37	−0.1618	0.0782	0.2460	0.079*	
C38	0.0086 (4)	0.0492 (3)	0.2173 (3)	0.0496 (12)	
H38	−0.0154	0.0129	0.1855	0.060*	

### Atomic displacement parameters (Å^2^)

**Table:**

	*U*^11^	*U*^22^	*U*^33^	*U*^12^	*U*^13^	*U*^23^
Co1	0.0380 (3)	0.0441 (3)	0.0307 (2)	−0.0055 (3)	−0.0038 (3)	0.0024 (3)
Cl1	0.0614 (9)	0.0627 (8)	0.0966 (11)	0.0083 (7)	−0.0118 (9)	−0.0187 (8)
O1	0.0477 (18)	0.0533 (18)	0.0398 (19)	−0.0136 (16)	−0.0067 (15)	0.0072 (14)
O2	0.0463 (17)	0.0517 (19)	0.0331 (16)	−0.0078 (15)	−0.0100 (13)	0.0051 (14)
O3	0.101 (3)	0.055 (2)	0.132 (4)	0.005 (2)	−0.002 (3)	−0.001 (2)
O4	0.127 (4)	0.087 (3)	0.121 (4)	0.035 (3)	0.032 (3)	−0.011 (3)
O5	0.057 (2)	0.098 (3)	0.124 (4)	−0.008 (2)	−0.002 (3)	0.012 (3)
O6	0.103 (4)	0.153 (4)	0.131 (4)	0.010 (3)	−0.049 (3)	−0.046 (4)
O7	0.098 (6)	0.118 (7)	0.047 (5)	0.066 (5)	−0.001 (4)	−0.044 (5)
O8	0.116 (7)	0.148 (8)	0.046 (5)	0.011 (6)	−0.024 (5)	0.004 (5)
O9	0.088 (7)	0.195 (11)	0.087 (7)	−0.027 (7)	−0.009 (5)	−0.009 (7)
N1	0.0372 (18)	0.0435 (19)	0.0358 (19)	−0.0038 (18)	−0.0029 (15)	−0.0018 (18)
N2	0.0333 (19)	0.044 (2)	0.032 (2)	−0.0011 (17)	−0.0010 (16)	−0.0020 (16)
N3	0.049 (2)	0.049 (2)	0.036 (2)	0.000 (2)	−0.0080 (19)	−0.0043 (17)
N4	0.039 (2)	0.049 (2)	0.034 (2)	−0.0047 (18)	0.0004 (17)	0.0069 (17)
C1	0.039 (2)	0.045 (2)	0.038 (3)	0.001 (2)	−0.005 (2)	0.004 (2)
C2	0.043 (3)	0.044 (2)	0.041 (3)	−0.003 (2)	−0.005 (2)	0.008 (2)
C3	0.048 (3)	0.061 (3)	0.058 (3)	−0.014 (3)	−0.009 (3)	0.017 (3)
C4	0.046 (3)	0.079 (4)	0.064 (4)	−0.019 (3)	−0.002 (3)	0.025 (3)
C5	0.047 (3)	0.076 (4)	0.058 (4)	−0.001 (3)	0.006 (3)	0.013 (3)
C6	0.046 (3)	0.056 (3)	0.044 (3)	0.000 (2)	−0.003 (2)	0.009 (2)
C7	0.042 (3)	0.048 (3)	0.034 (3)	0.001 (2)	−0.007 (2)	0.004 (2)
C8	0.043 (3)	0.051 (3)	0.032 (3)	0.000 (2)	0.0064 (19)	0.002 (2)
C9	0.040 (3)	0.054 (3)	0.027 (2)	0.001 (2)	0.0042 (19)	−0.002 (2)
C10	0.040 (3)	0.070 (3)	0.034 (3)	0.003 (3)	0.000 (2)	0.002 (2)
C11	0.042 (3)	0.083 (4)	0.037 (3)	0.013 (3)	−0.001 (2)	0.011 (3)
C12	0.050 (3)	0.062 (3)	0.042 (3)	0.011 (3)	0.005 (2)	0.017 (2)
C13	0.051 (3)	0.054 (3)	0.037 (3)	0.004 (2)	0.005 (2)	0.009 (2)
C14	0.041 (3)	0.046 (3)	0.036 (3)	−0.007 (2)	0.002 (2)	0.000 (2)
C15	0.032 (2)	0.047 (3)	0.039 (3)	−0.0034 (19)	−0.0050 (19)	−0.001 (2)
C16	0.035 (2)	0.043 (2)	0.035 (2)	−0.005 (2)	−0.0020 (19)	−0.002 (2)
C17	0.038 (2)	0.049 (3)	0.043 (3)	−0.006 (2)	−0.008 (2)	0.007 (2)
C18	0.044 (3)	0.066 (3)	0.062 (3)	−0.006 (3)	−0.007 (2)	0.006 (3)
C19	0.054 (3)	0.094 (4)	0.076 (4)	−0.005 (4)	−0.020 (3)	0.013 (4)
C20	0.069 (4)	0.096 (5)	0.071 (4)	−0.025 (4)	−0.028 (3)	0.023 (4)
C21	0.087 (4)	0.078 (4)	0.061 (4)	−0.008 (3)	−0.021 (4)	−0.003 (3)
C22	0.063 (3)	0.068 (3)	0.045 (3)	0.004 (3)	−0.016 (3)	−0.002 (3)
C23	0.035 (2)	0.050 (3)	0.040 (3)	0.001 (2)	0.001 (2)	0.002 (2)
C24	0.056 (3)	0.070 (3)	0.057 (3)	−0.011 (3)	0.012 (3)	−0.013 (3)
C25	0.052 (3)	0.087 (4)	0.067 (3)	−0.006 (3)	0.022 (3)	−0.012 (3)
C26	0.040 (3)	0.075 (4)	0.066 (4)	−0.006 (3)	0.011 (2)	0.014 (3)
C27	0.048 (3)	0.052 (3)	0.064 (3)	−0.005 (3)	0.005 (3)	0.011 (2)
C28	0.040 (3)	0.044 (2)	0.047 (3)	−0.001 (2)	0.001 (2)	0.004 (2)
C29	0.073 (4)	0.060 (3)	0.051 (3)	0.010 (3)	−0.010 (3)	−0.006 (3)
C30	0.111 (5)	0.077 (4)	0.075 (5)	0.037 (4)	−0.014 (4)	−0.014 (4)
C31	0.093 (5)	0.093 (5)	0.075 (5)	0.033 (4)	−0.002 (4)	−0.028 (4)
C32	0.088 (5)	0.082 (4)	0.051 (3)	0.010 (4)	0.005 (3)	−0.010 (3)
C33	0.061 (3)	0.058 (3)	0.045 (3)	−0.003 (3)	0.002 (3)	−0.012 (2)
C34	0.040 (3)	0.060 (3)	0.047 (3)	−0.002 (2)	0.006 (2)	−0.002 (2)
C35	0.066 (4)	0.069 (3)	0.059 (4)	0.010 (3)	0.009 (3)	−0.013 (3)
C36	0.055 (4)	0.088 (4)	0.076 (4)	0.016 (3)	0.016 (3)	0.003 (4)
C37	0.041 (3)	0.093 (4)	0.064 (4)	0.007 (3)	0.008 (3)	0.013 (3)
C38	0.041 (3)	0.061 (3)	0.047 (3)	−0.003 (2)	−0.002 (2)	0.009 (2)

### Geometric parameters (Å, °)

**Table:**

Co1—O2	1.881 (3)	C13—H13	0.9300
Co1—O1	1.889 (3)	C14—H14	0.9300
Co1—N2	1.897 (3)	C15—C16	1.509 (6)
Co1—N1	1.904 (3)	C15—C17	1.534 (6)
Co1—N4	1.973 (4)	C15—H15	0.9800
Co1—N3	1.978 (4)	C16—C23	1.524 (6)
Cl1—O6	1.400 (5)	C16—H16	0.9800
Cl1—O5	1.412 (4)	C17—C18	1.376 (6)
Cl1—O4	1.424 (4)	C17—C22	1.387 (6)
Cl1—O3	1.456 (4)	C18—C19	1.383 (7)
O1—C2	1.329 (5)	C18—H18	0.9300
O2—C9	1.318 (5)	C19—C20	1.369 (8)
O7—H7C	0.8501	C19—H19	0.9300
O7—H7D	0.8499	C20—C21	1.393 (8)
O8—H8C	0.8500	C20—H20	0.9300
O8—H8D	0.8500	C21—C22	1.386 (7)
O9—H9C	0.8500	C21—H21	0.9300
O9—H9D	0.8500	C22—H22	0.9300
N1—C7	1.290 (5)	C23—C24	1.374 (6)
N1—C15	1.482 (5)	C23—C28	1.385 (6)
N2—C14	1.277 (5)	C24—C25	1.400 (7)
N2—C16	1.508 (5)	C24—H24	0.9300
N3—C29	1.328 (6)	C25—C26	1.359 (7)
N3—C33	1.342 (6)	C25—H25	0.9300
N4—C34	1.332 (5)	C26—C27	1.366 (7)
N4—C38	1.351 (5)	C26—H26	0.9300
C1—C6	1.395 (6)	C27—C28	1.398 (6)
C1—C2	1.418 (6)	C27—H27	0.9300
C1—C7	1.436 (6)	C28—H28	0.9300
C2—C3	1.401 (6)	C29—C30	1.361 (8)
C3—C4	1.343 (7)	C29—H29	0.9300
C3—H3	0.9300	C30—C31	1.380 (9)
C4—C5	1.383 (7)	C30—H30	0.9300
C4—H4	0.9300	C31—C32	1.345 (9)
C5—C6	1.378 (7)	C31—H31	0.9300
C5—H5	0.9300	C32—C33	1.385 (7)
C6—H6	0.9300	C32—H32	0.9300
C7—H7	0.9300	C33—H33	0.9300
C8—C9	1.408 (6)	C34—C35	1.381 (6)
C8—C13	1.414 (6)	C34—H34	0.9300
C8—C14	1.459 (6)	C35—C36	1.370 (8)
C9—C10	1.412 (6)	C35—H35	0.9300
C10—C11	1.386 (7)	C36—C37	1.369 (8)
C10—H10	0.9300	C36—H36	0.9300
C11—C12	1.379 (7)	C37—C38	1.376 (7)
C11—H11	0.9300	C37—H37	0.9300
C12—C13	1.365 (7)	C38—H38	0.9300
C12—H12	0.9300		
			
O2—Co1—O1	87.26 (12)	C8—C14—H14	117.5
O2—Co1—N2	95.39 (13)	N1—C15—C16	107.3 (3)
O1—Co1—N2	177.35 (14)	N1—C15—C17	115.0 (3)
O2—Co1—N1	179.34 (15)	C16—C15—C17	111.6 (3)
O1—Co1—N1	93.06 (13)	N1—C15—H15	107.6
N2—Co1—N1	84.29 (15)	C16—C15—H15	107.6
O2—Co1—N4	88.04 (14)	C17—C15—H15	107.6
O1—Co1—N4	88.81 (14)	N2—C16—C15	108.3 (3)
N2—Co1—N4	91.41 (14)	N2—C16—C23	114.4 (3)
N1—Co1—N4	92.55 (14)	C15—C16—C23	112.6 (4)
O2—Co1—N3	86.52 (14)	N2—C16—H16	107.1
O1—Co1—N3	88.97 (15)	C15—C16—H16	107.1
N2—Co1—N3	91.06 (15)	C23—C16—H16	107.1
N1—Co1—N3	92.90 (15)	C18—C17—C22	119.0 (4)
N4—Co1—N3	174.22 (16)	C18—C17—C15	120.3 (4)
O6—Cl1—O5	110.2 (3)	C22—C17—C15	120.6 (4)
O6—Cl1—O4	109.1 (3)	C17—C18—C19	120.6 (5)
O5—Cl1—O4	109.5 (3)	C17—C18—H18	119.7
O6—Cl1—O3	109.1 (3)	C19—C18—H18	119.7
O5—Cl1—O3	109.8 (3)	C20—C19—C18	120.3 (6)
O4—Cl1—O3	109.0 (3)	C20—C19—H19	119.8
C2—O1—Co1	121.9 (3)	C18—C19—H19	119.8
C9—O2—Co1	123.9 (3)	C19—C20—C21	120.0 (6)
H7C—O7—H7D	108.5	C19—C20—H20	120.0
H8C—O8—H8D	108.3	C21—C20—H20	120.0
H9C—O9—H9D	108.8	C22—C21—C20	119.2 (6)
C7—N1—C15	123.1 (3)	C22—C21—H21	120.4
C7—N1—Co1	124.3 (3)	C20—C21—H21	120.4
C15—N1—Co1	112.5 (3)	C21—C22—C17	120.8 (5)
C14—N2—C16	119.3 (4)	C21—C22—H22	119.6
C14—N2—Co1	124.2 (3)	C17—C22—H22	119.6
C16—N2—Co1	115.0 (2)	C24—C23—C28	119.3 (4)
C29—N3—C33	117.5 (4)	C24—C23—C16	122.0 (4)
C29—N3—Co1	122.5 (3)	C28—C23—C16	118.6 (4)
C33—N3—Co1	119.7 (3)	C23—C24—C25	120.1 (5)
C34—N4—C38	118.3 (4)	C23—C24—H24	119.9
C34—N4—Co1	124.4 (3)	C25—C24—H24	119.9
C38—N4—Co1	117.3 (3)	C26—C25—C24	119.9 (5)
C6—C1—C2	119.7 (4)	C26—C25—H25	120.0
C6—C1—C7	117.9 (4)	C24—C25—H25	120.0
C2—C1—C7	122.3 (4)	C25—C26—C27	120.9 (5)
O1—C2—C3	120.3 (4)	C25—C26—H26	119.5
O1—C2—C1	122.7 (4)	C27—C26—H26	119.5
C3—C2—C1	117.0 (4)	C26—C27—C28	119.6 (5)
C4—C3—C2	121.9 (5)	C26—C27—H27	120.2
C4—C3—H3	119.1	C28—C27—H27	120.2
C2—C3—H3	119.1	C23—C28—C27	120.1 (4)
C3—C4—C5	121.9 (5)	C23—C28—H28	119.9
C3—C4—H4	119.1	C27—C28—H28	119.9
C5—C4—H4	119.1	N3—C29—C30	123.5 (6)
C6—C5—C4	118.3 (5)	N3—C29—H29	118.2
C6—C5—H5	120.9	C30—C29—H29	118.2
C4—C5—H5	120.9	C29—C30—C31	118.6 (6)
C5—C6—C1	121.2 (4)	C29—C30—H30	120.7
C5—C6—H6	119.4	C31—C30—H30	120.7
C1—C6—H6	119.4	C32—C31—C30	119.0 (6)
N1—C7—C1	124.4 (4)	C32—C31—H31	120.5
N1—C7—H7	117.8	C30—C31—H31	120.5
C1—C7—H7	117.8	C31—C32—C33	119.8 (6)
C9—C8—C13	120.2 (4)	C31—C32—H32	120.1
C9—C8—C14	122.2 (4)	C33—C32—H32	120.1
C13—C8—C14	117.5 (4)	N3—C33—C32	121.6 (5)
O2—C9—C8	125.1 (4)	N3—C33—H33	119.2
O2—C9—C10	117.2 (4)	C32—C33—H33	119.2
C8—C9—C10	117.7 (4)	N4—C34—C35	122.8 (5)
C11—C10—C9	120.6 (5)	N4—C34—H34	118.6
C11—C10—H10	119.7	C35—C34—H34	118.6
C9—C10—H10	119.7	C36—C35—C34	118.8 (5)
C12—C11—C10	121.0 (5)	C36—C35—H35	120.6
C12—C11—H11	119.5	C34—C35—H35	120.6
C10—C11—H11	119.5	C37—C36—C35	118.7 (5)
C13—C12—C11	119.9 (5)	C37—C36—H36	120.7
C13—C12—H12	120.0	C35—C36—H36	120.7
C11—C12—H12	120.0	C36—C37—C38	120.3 (5)
C12—C13—C8	120.5 (5)	C36—C37—H37	119.9
C12—C13—H13	119.8	C38—C37—H37	119.9
C8—C13—H13	119.8	N4—C38—C37	121.1 (5)
N2—C14—C8	125.0 (4)	N4—C38—H38	119.4
N2—C14—H14	117.5	C37—C38—H38	119.4

### Hydrogen-bond geometry (Å, °)

**Table:**

*D*—H···*A*	*D*—H	H···*A*	*D*···*A*	*D*—H···*A*
O7—H7C···O8	0.85	1.85	2.701 (12)	176
O7—H7D···O1^i^	0.85	2.04	2.888 (8)	176
O8—H8C···O9	0.85	1.73	2.575 (14)	177
O8—H8D···O4^ii^	0.85	1.99	2.835 (10)	176
O9—H9C···O3^ii^	0.85	2.36	3.175 (11)	161

Symmetry codes: (i) *x*+1/2, −*y*+1/2, −*z*+1; (ii) −*x*+1/2, −*y*+1, *z*+1/2.
